# A Study on Electro-Assisted Forming of Thin-Walled Skin Components Made of Ti-6Al-4V Alloy

**DOI:** 10.3390/ma19153352

**Published:** 2026-08-06

**Authors:** Zhengang Yuan, Xuefeng Xu, Jiaqi Huang, Jun Xie, Fengwei Zhang, Kai Tian

**Affiliations:** 1 School of Mechanical and Electrical Engineering, Nanchang Hangkong University, Nanchang 330063, China; 19979076272@163.com (Z.Y.); 15727584182@163.com (J.H.); 71000@nchu.edu.cn (J.X.); 19705306616@163.com (F.Z.); 2 Shaanxi Aircraft Industry Co., Ltd., Hanzhong 723200, China; 15879026794@163.com

**Keywords:** Ti-6Al-4V alloy, constitutive model, electrically assisted forming, microstructure

## Abstract

To address the challenges of procedural complexity, the lack of an integrated heating–forming capability, and poor formability in the hot forming of titanium alloy skin components, an electrically assisted forming (EAF) process is proposed. A Johnson–Cook constitutive model was established to characterize the flow behavior of Ti–6Al–4V alloy under electric-assisted conditions, achieving a correlation coefficient of 0.968 and an average relative error of 7.67%. Forming parameters were investigated through a combined approach of numerical simulation and experimentation. At a current density of 7.59 A/mm^2^, a forming speed of 1 mm/min, and a friction coefficient of 0.1, the maximum springback of the component was 1.04 mm. Compared with isothermal forming, the EAF process reduced the springback by 7.14% and enhanced the ultimate tensile strength by 5.34%. Microstructural characterization revealed that, under pulsed current, the α-phase grains of the material were refined, whereas the β-phase fraction and the average grain size increased, accompanied by a 15.3% reduction in the geometrically necessary dislocation (GND) density. This study validates the process feasibility of electrically assisted forming for thin-walled titanium alloy skin components.

## 1. Introduction

Titanium alloys have become indispensable structural materials in advanced equipment manufacturing sectors such as aerospace, owing to their light weight, excellent specific strength, and superior corrosion resistance. They are widely employed in critical components including aircraft skins, trusses, and engine parts [1,2,3]. With the ongoing advancement of aerospace equipment toward lightweight construction, high precision, and enhanced reliability, increasingly stringent demands are being placed on both the forming quality and production efficiency of titanium alloy components. However, titanium alloys exhibit poor formability and high yield strength at room temperature. To overcome these challenges, isothermal forming (IF) has conventionally been adopted to reduce the deformation resistance of the material. Nevertheless, this approach suffers from several inherent drawbacks, including low heating efficiency, severe oxidation and hydrogenation of the workpiece, and poor forming accuracy [4,5], which collectively impede the high-quality and high-efficiency production of complex-shaped parts. Electrically assisted forming (EAF) has emerged as a promising alternative to conventional thermoplastic forming processes, offering distinct advantages such as high heating efficiency, low energy consumption, and the integration of heating and forming into a single operation [6,7,8]. By introducing a pulsed current during plastic deformation, EAF exploits the Joule heating effect to rapidly heat the material, thereby reducing the flow stress and enhancing ductility. Concurrently, the electroplastic effect further improves the formability of difficult-to-deform materials [9,10,11].

Extensive research has been conducted on the deformation behavior, constitutive modeling, and microstructural evolution of titanium alloys under electrically assisted conditions. Qiao et al. [12] developed an electro-thermo-mechanical coupled constitutive model for Ti-6Al-4V alloy based on the Johnson-Cook framework. By incorporating thermal and electrical effect functions, their model effectively captures the stress–strain response of the alloy under electric-assisted deformation. Bao et al. [13] conducted electrically assisted tensile tests and reported that the flow stress of Ti-6Al-4V alloy decreased progressively with increasing current density and pulse width. Through a material flow model, they estimated that approximately 11% of the stress reduction originated from athermal effects of the electric current, while attributing the primary reduction to Joule-heating-induced softening and thermal expansion. In a subsequent study, Bao et al. [14] performed electrically assisted micro-compression (EAMC) experiments on titanium alloys. Microstructural observations revealed that the pulsed current induced an α-phase transformation sequence of α → β → α′ in Ti-6Al-4V alloy.

To date, extensive research efforts have been devoted to electrically assisted forming processes. Bao et al. [15] implemented electrically assisted incremental forming of AZ31B alloy using a computer numerical control (CNC) machine tool combined with a pulsed power supply. They reported that when the current density reached 107.8 A/mm^2^, the forming limit angle of the material increased from 39.6° (without current application) to 72°. Microstructural characterization revealed that the pulsed current promoted dynamic recrystallization, suppressed crack propagation, and consequently enhanced the formability of the components. Li et al. [16] developed an electrically assisted gas blow forming process by replacing conventional furnace heating with electrical heating in the gas blow forming technique. By improving the temperature distribution of the sheet blank via electrical heating, they achieved a substantial enhancement in part formability. The introduction of electric current nearly doubled the forming efficiency, while reducing energy consumption by 27.5% compared with conventional hot forming.

Furthermore, electrically assisted forming has demonstrated a pronounced capability in suppressing springback during bending operations. Meng et al. [17] investigated the current-assisted V-bending process of Ti–6Al–4V alloy sheets and found that the application of pulsed current enhanced the bending limit of the material, while both the bending springback angle and the forming force decreased progressively with increasing current density. Chu et al. [18] examined the V-bending behavior of AZ31B magnesium alloy under pulsed current assistance. They reported that at a current density of 13.02 A/mm^2^ and a frequency of 350 Hz, the forming force was reduced by 43.31% and the springback angle was decreased by 18.57% compared with the current-free condition. In comparison with conventional hot forming processes, they attributed the enhanced formability primarily to the athermal effects of the electric current, which promoted dynamic recrystallization. Li et al. [19] developed a pulsed-current-assisted stamping method tailored for ultrathin metallic sheets. In their approach, the sheet was first pierced, and the subsequent application of pulsed current served to mitigate the springback induced by the piercing operation, thereby substantially improving the quality of the formed parts. They observed that a pulsed current with a density of 26.56 A/mm^2^ and a frequency of 1 Hz suppressed 83% of the channel-height loss attributable to springback. In addition, electrically assisted forming has also shown significant advantages in other manufacturing processes, including rolling, turning, and welding [20,21,22,23].

In summary, existing studies on electrically assisted forming of Ti-6Al-4V alloy have predominantly focused on fundamental material properties. However, the applicability and forming quality of EAF for aerospace components characterized by complex curved surfaces, large dimensions, and thin-walled features remain insufficiently investigated. To address this gap, the present study proposes an electrically assisted forming process for manufacturing thin-walled Ti-6Al-4V alloy skin components. Through a combined approach of finite element simulation and experimentation, the effects of various forming conditions on the formability of Ti-6Al-4V alloy sheets were systematically investigated. Comparative experiments between electrically assisted forming and isothermal forming were conducted, followed by mechanical testing and microstructural characterization of the formed components. The findings are expected to provide a theoretical basis and technical guidance for the electrically assisted forming of thin-walled titanium alloy components.

## 2. Experimental Components and Materials

### 2.1. Experimental Components

**Figure 1:**
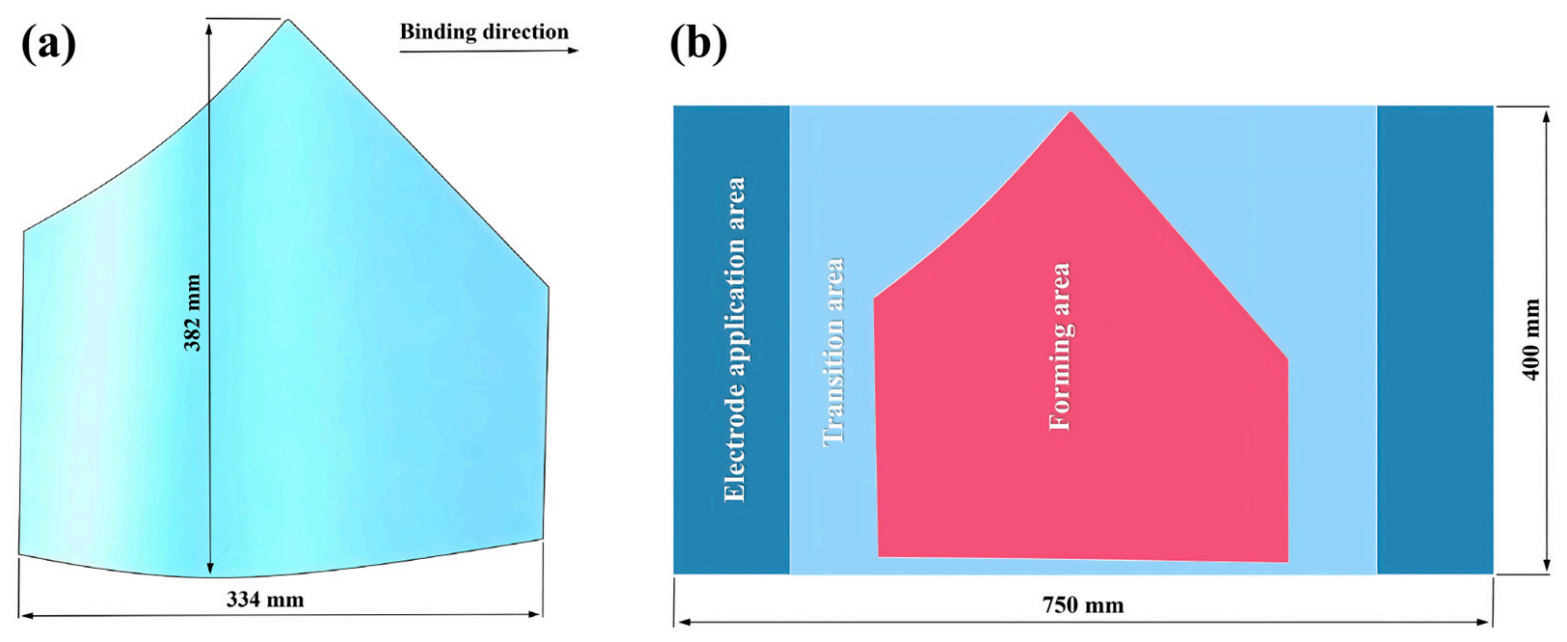
(**a**) Ti-6Al-4V alloy skin components; (**b**) rectangular billet.

The titanium alloy sheet metal part studied in this paper is shown in Figure 1a. This part is a thin-walled skin component, with bending deformation as its primary deformation mode, and has basic dimensions of 334 mm × 382 mm. The part was reverse-unfolded and expanded into a rectangular blank measuring 750 mm × 400 mm, as shown in Figure 1b. The blank was designed with sufficient length to accommodate the electrodes.

### 2.2. Materials and Equipment

**Figure 2:**
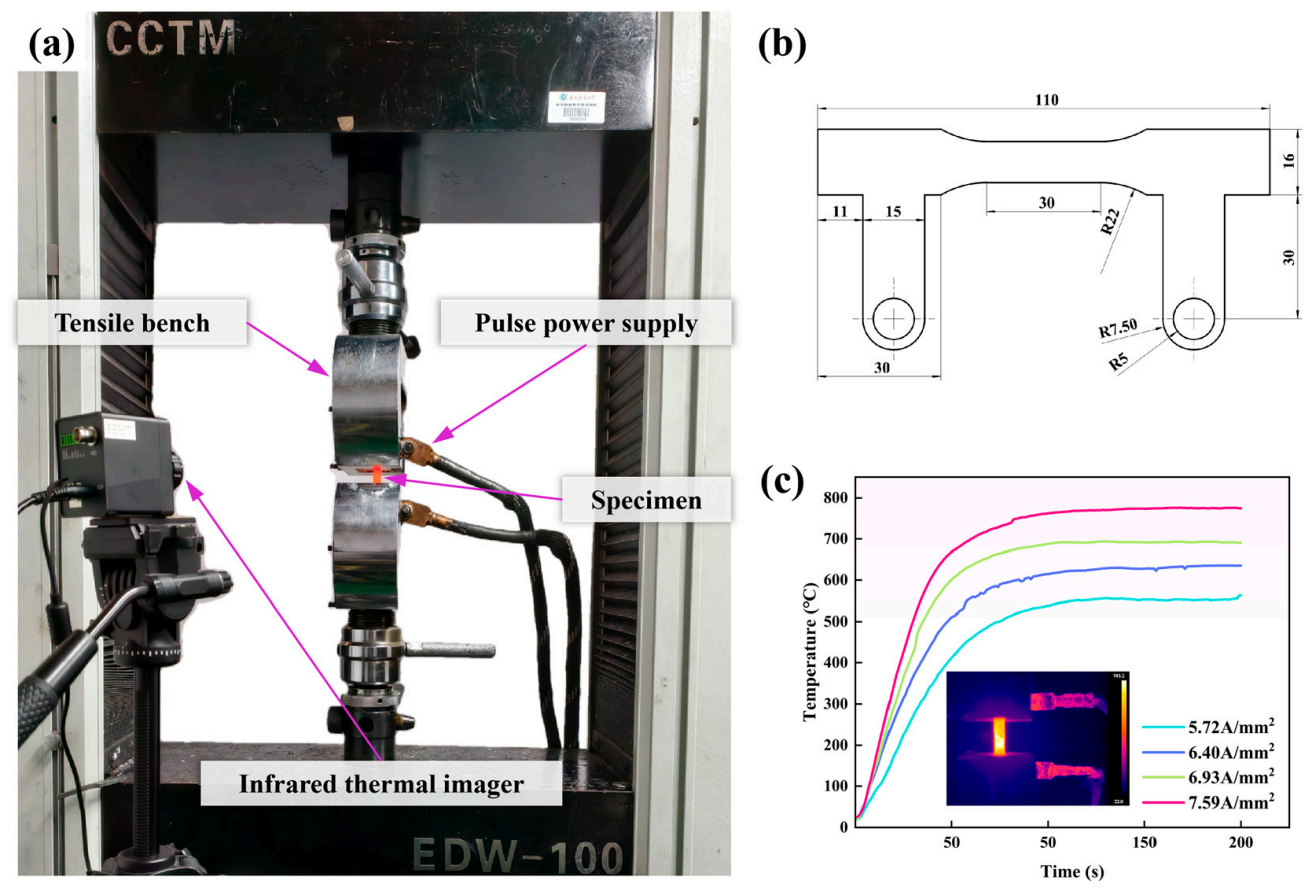
Experimental setup for electric pulse-assisted tensile testing: (**a**) Experimental setup; (**b**) dimensions of the tensile specimen; (**c**) heating curves at different current densities.

The material used in the experiment was a 1.5-mm-thick Ti-6Al-4V alloy plate; its chemical composition by mass fraction is shown in Table 1. Electrically assisted tensile specimens were prepared along the rolling direction, with lugs added to apply an electrical load; the specimen geometry is shown in Figure 2b.

An electrically assisted tensile testing platform was constructed using an EDW-100 universal testing machine (Changchun New Testing Machine Co., Ltd., Changchun, China), as shown in Figure 2a. A HNZM100-M2500 programmable pulse power supply (Shandong Hangneng Electrical Equipment Co., Ltd., Jinan, China) was used to apply a pulse current to the specimen, with a current output range of 0–5000 A. Real-time temperature measurements were taken using an infrared thermal imager (X384D1600MF15) (Yoseen Infrared, Wuhan, China), which has a temperature measurement range of –20 °C to 1600 °C and a measurement accuracy of ±2 °C. During the tests, the duty cycle was 20% and the pulse frequency was 100 Hz. Tests were conducted at current densities of 5.72 A/mm^2^, 6.40 A/mm^2^, 6.93 A/mm^2^, and 7.59 A/mm^2^ at strain rates of 0.001 s^−1^, 0.005 s^−1^, 0.01 s^−1^, and 0.05 s^−1^. The corresponding temperatures were 552 °C, 635 °C, 696 °C, and 788 °C. As shown in Figure 2c, in the electrically pulsed tensile test, an electrical load was applied to the tensile specimen, followed by a 150-s current preheating phase. Once the temperature stabilized, tensile testing was conducted, with each set of experiments repeated at least three times to ensure reliability.

## 3. Mechanical Properties and Constitutive Equations

### 3.1. Stress–Strain Curve

**Figure 3:**
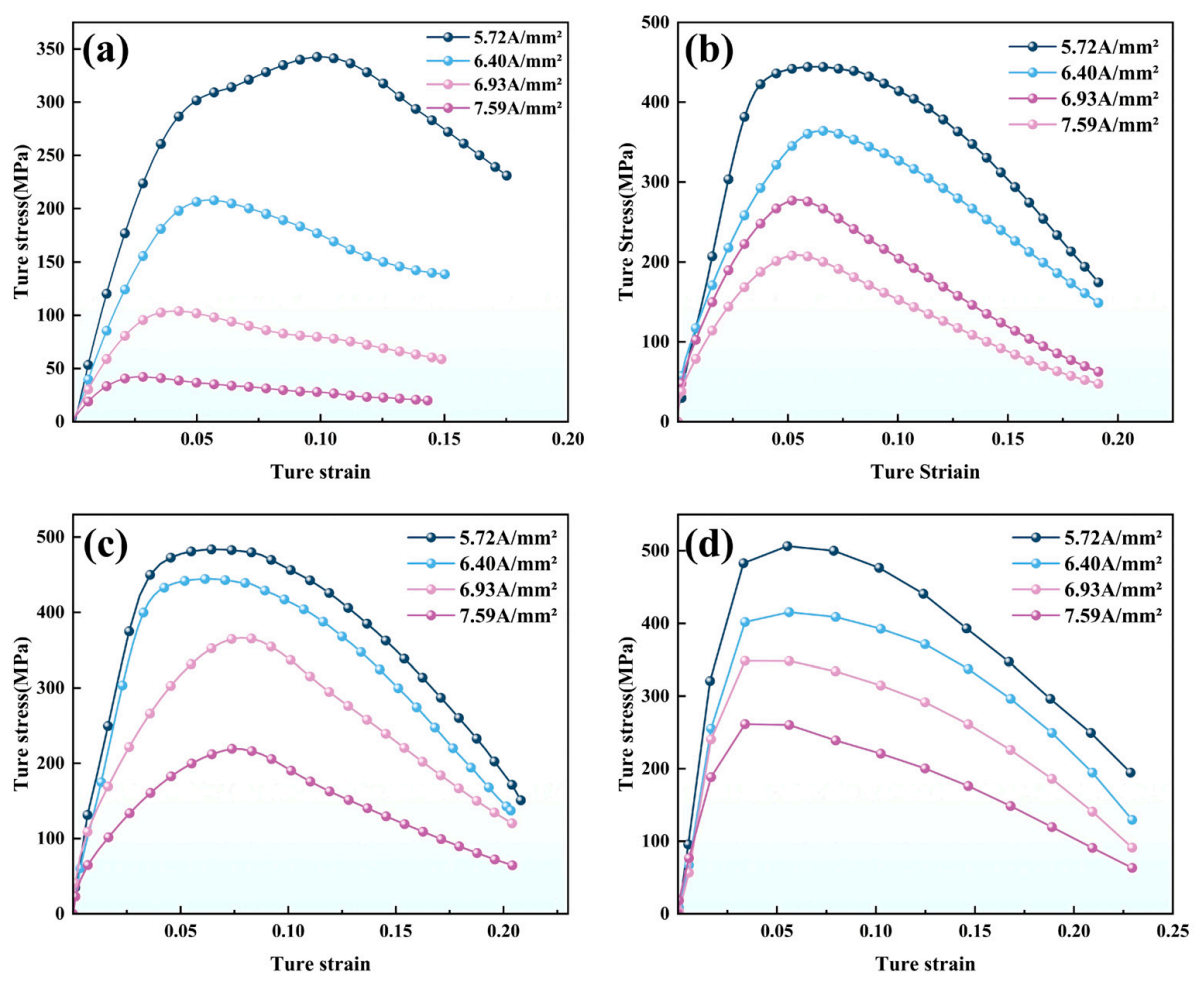
True stress–strain curve for electric pulse-assisted tensile testing: (**a**) 0.001 s^−1^; (**b**) 0.005 s^−1^; (**c**) 0.01 s^−1^; (**d**) 0.05 s^−1^.

The experimental results of electrically assisted tensile tests on Ti-6Al-4V alloy under different current densities and deformation speeds are presented in Figure 3. At a given deformation speed, the flow stress of the material decreased progressively with increasing current density. Conversely, at a fixed current density, the flow stress increased with increasing deformation speed.

### 3.2. Constitutive Model

The Johnson-Cook model is used to characterize the stress–strain constitutive relationship of the Ti-6Al-4V alloy under electrically assisted stretching, as expressed by the following equation [24]:

(1) $$\sigma =(A+B\varepsilon^{n})(1+\mathrm{Cln}\frac{\dot{\varepsilon }}{{\dot{\varepsilon }}_{0}})[1-(\frac{T-T_{R}}{T_{m}-T_{R}}{)}^{m}]$$

In the equation: σ is the equivalent flow stress; ε is the true strain; A is the yield stress of the material at the reference strain rate; B is the strain-hardening modulus of the material; n is the strain-hardening exponent of the material; C is the strain rate strengthening coefficient of the material; m is the thermal softening coefficient of the material; $\dot{\varepsilon \;}$ is the equivalent strain rate; ${\dot{\varepsilon }}_{0}$ is the reference strain rate; $T_{R}$ is the reference temperature; $T_{m}$ is the melting point of the material; T is the temperature of the material.

This study is based on the modified J-C model proposed by Lin Y C et al. [25], in which polynomials were added to both the linear strain component and the logarithmic strain rate term to accurately represent the effects of strain and strain rate on true stress. The model is as follows:

(2) $$\sigma =A(\varepsilon )C(\ln{\dot{\varepsilon }}^{*})\exp[\lambda (\ln{\dot{\varepsilon }}^{*})(T-T_{r})]$$

$$A(\varepsilon )=A_{1}+B_{1}\varepsilon +B_{2}\varepsilon^{2}$$

(3) $$C(\ln{\dot{\varepsilon }}^{*})=C_{0}+C_{1}\ln{\dot{\varepsilon }}^{*}+C_{2}\ln{\dot{\varepsilon }}^{*2}$$

$$\lambda (\ln{\dot{\varepsilon }}^{*})=\lambda_{0}+\lambda_{1}\ln{\dot{\varepsilon }}^{*}+\lambda_{2}\ln{\dot{\varepsilon }}^{*2}+\lambda_{3}\ln{\dot{\varepsilon }}^{*3}$$

The reference temperature $T_{r}$ is the temperature corresponding to a current density of 6.40 A/mm^2^, and the reference strain rate is 0.001 s^−1^; Equation (2) can then be rewritten as:

(4) $$\sigma =A_{1}+B_{1}\varepsilon +B_{2}\varepsilon^{2}$$

**Figure 4:**
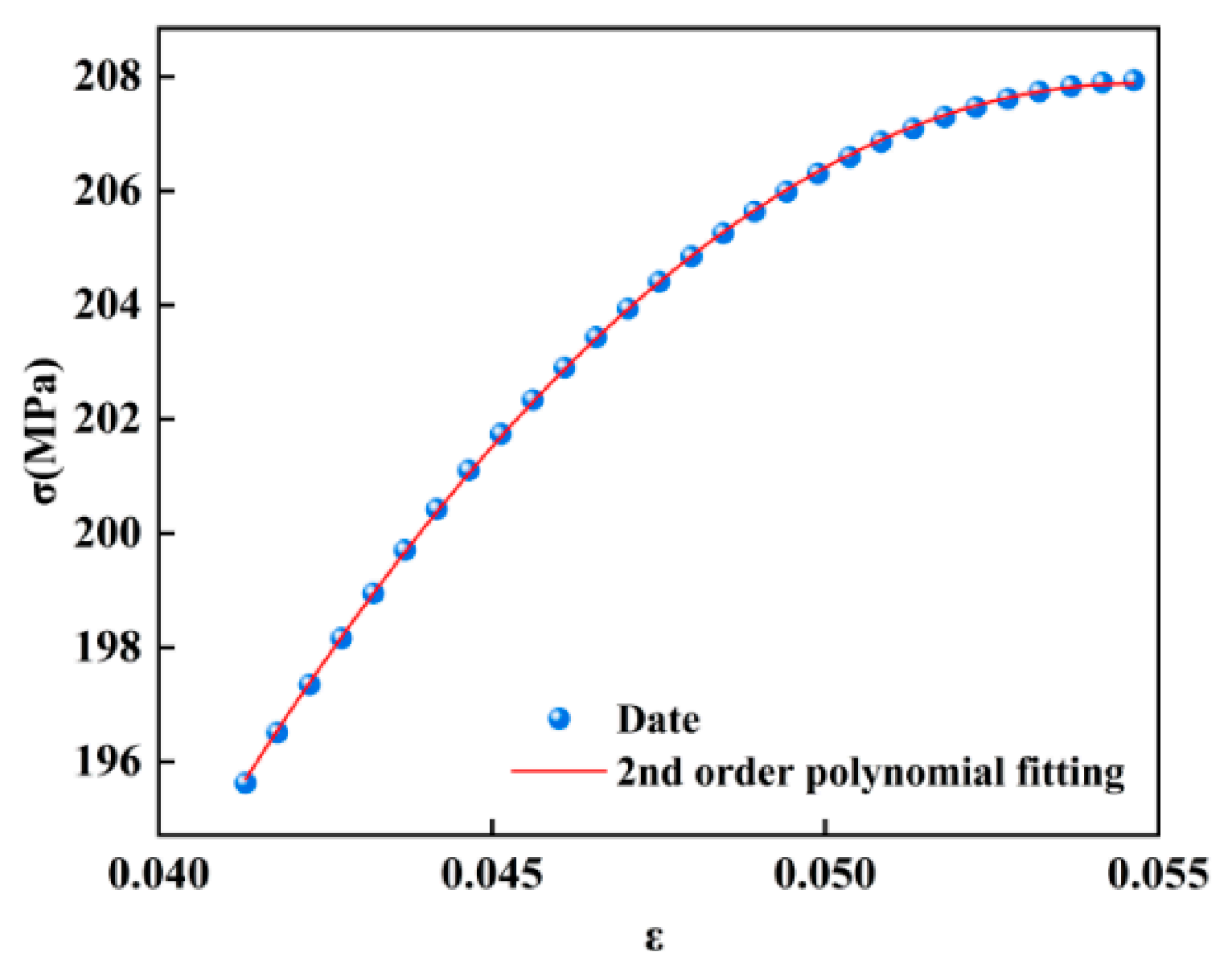
Quadratic polynomial fit of the σ−ε curve.

A quadratic polynomial fit was performed on the scatter plot; the results are shown in Figure 4. The values of A_1_, B_1_, and B_2_ can be estimated from the stress–strain data as A_1_ = 2.9453, B_1_ = 7503.94, and B_2_ = −68693.54, respectively.

Equation (4) can be expressed as:

(5) $$\sigma =2.9453+7503.94\varepsilon {-68693.54\varepsilon }^{2}$$

At the reference temperature, Equation (2) can be rewritten as:

(6) $$\frac{\sigma }{A_{1}+B_{1}\varepsilon +B_{2}\varepsilon^{2}}=C_{0}+C_{1}\ln{\dot{\varepsilon }}^{*}+C_{2}\ln{\dot{\varepsilon }}^{*2}$$

**Figure 5:**
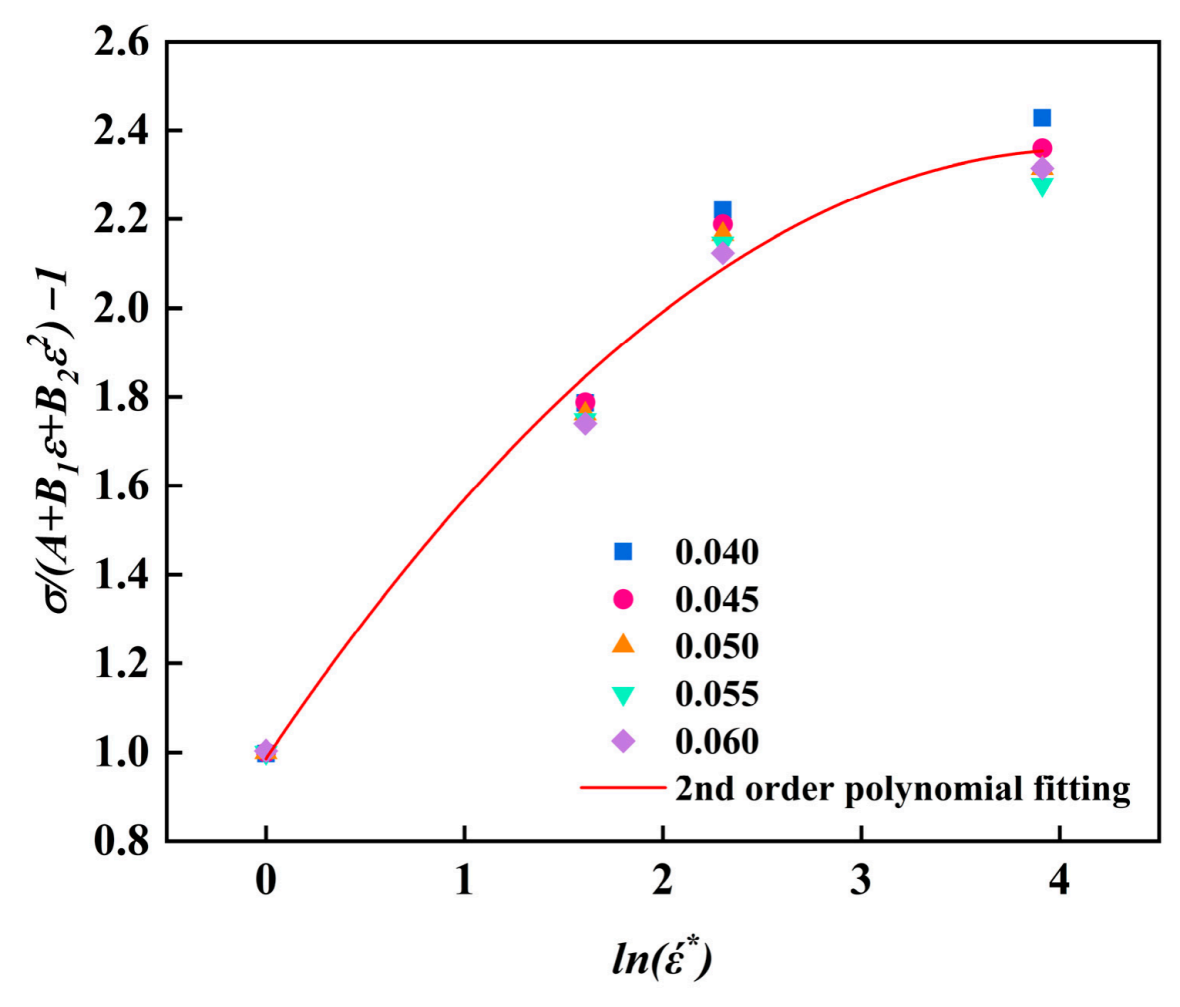
Quadratic polynomial fit curves for $[\sigma /(A_{1}\;+\;B_{1}\varepsilon \;+\;B_{2}\varepsilon^{2})-$ 1] − $\ln({\dot{\varepsilon }}^{*})$ at different strains.

Under different strain rate conditions, a scatter plot of $\frac{\sigma }{A_{1}+B_{1}\varepsilon +B_{2}\varepsilon^{2}}-\ln({\dot{\varepsilon }}^{*})$ was plotted and fitted using a quadratic polynomial, as shown in Figure 5, yielding C_0_ = 0.98866, C_1_ = 0.66288, and C_2_ = −0.0801.

Introducing a new parameter λ, where $\lambda =\lambda_{0}+\lambda_{1}\ln{\dot{\varepsilon }}^{*}+\lambda_{2}\ln{\dot{\varepsilon }}^{*2}+\lambda_{3}\ln{\dot{\varepsilon }}^{*3}$, Equation (2) can be expressed as follows:

(7) $$\frac{\sigma }{\left[{(A}_{1}+B_{1}\varepsilon +B_{2}\varepsilon^{2})\times (C_{0}+C_{1}\ln{\dot{\varepsilon }}^{*}+C_{2}\ln{\dot{\varepsilon }}^{*2})\right]}=e^{\lambda (T-T_{r})}$$

Taking the natural logarithm of both sides of Equation (7) yields:

(8) $$\ln\left\{\frac{\sigma }{\left[{(A}_{1}+B_{1}\varepsilon +B_{2}\varepsilon^{2})\times (C_{0}+C_{1}\ln{\dot{\varepsilon }}^{*}+C_{2}\ln{\dot{\varepsilon }}^{*2})\right]\;}\right\}=\lambda (T-T_{r})$$

**Figure 6:**
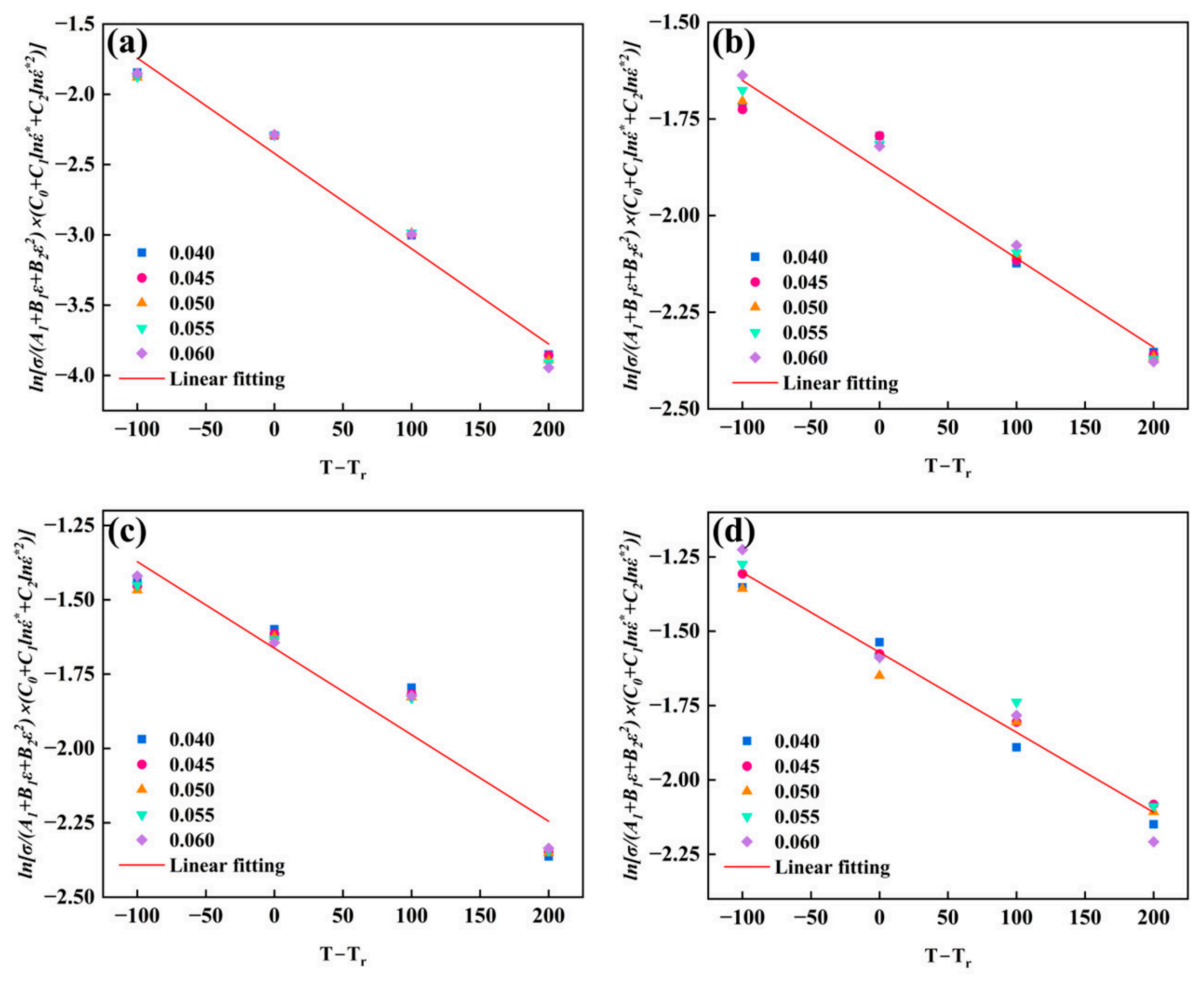
Linear fitting curves for $\ln\left\{\sigma /\left[{(A}_{1}+B_{1}\varepsilon +B_{2}\varepsilon^{2})\times (C_{0}+C_{1}\ln{\dot{\varepsilon }}^{*}\;+{\;C}_{2}\ln{\dot{\varepsilon }}^{*2})\right]\right\}-(T-T_{r})$ at different strains: (**a**) ${\dot{\varepsilon }}^{*}=1$; (**b**) ${\dot{\varepsilon }}^{*}=5$; (**c**) ${\dot{\varepsilon }}^{*}=10$; (**d**) ${\dot{\varepsilon }}^{*}=50$.

**Figure 7:**
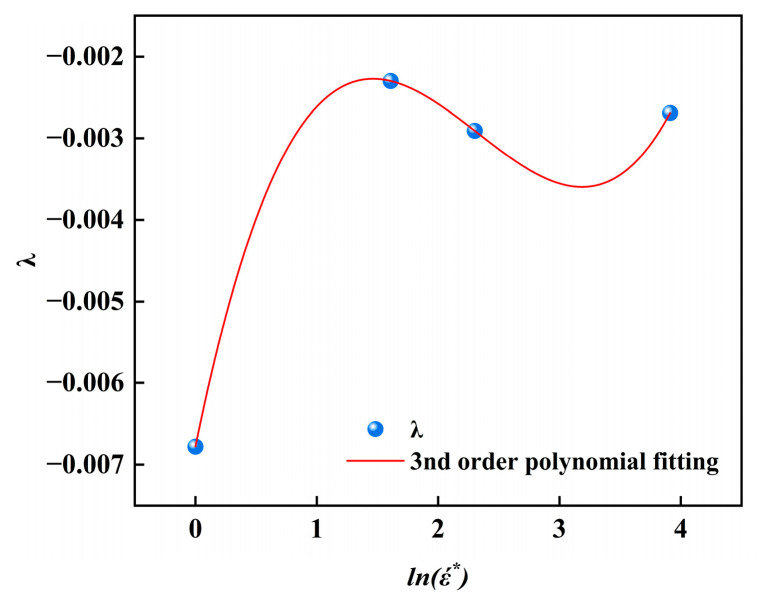
Relationship between $\lambda \;-\;\ln{\dot{\varepsilon }}^{*}.$

For different strain rates, strains, and deformation temperatures, the relationship between $\ln\left\{\sigma /\left[{(A}_{1}+B_{1}\varepsilon +B_{2}\varepsilon^{2})\times (C_{0}+C_{1}\ln{\dot{\varepsilon }}^{*}+C_{2}\ln{\dot{\varepsilon }}^{*2})\right]\right\}$ and $(T-T_{r})$ can be obtained, as shown in Figure 6. For $\lambda_{\left({\dot{\varepsilon }}^{*}=\;1\right)}$, $\lambda_{\left({\dot{\varepsilon }}^{*}=\;5\right)}$, $\lambda_{\left({\dot{\varepsilon }}^{*}=\;10\right)}$, and $\lambda_{\left({\dot{\varepsilon }}^{*}=\;50\right)}$, the slopes obtained via linear fitting are −0.00678, −0.0023, −0.00291, and −0.00269, respectively. After fitting the $\lambda \;-\;\ln{\dot{\varepsilon }}^{*}$ data with a cubic polynomial, the coefficients $\lambda_{0}$, $\lambda_{1}$, $\lambda_{2}$, and $\lambda_{3}$ were determined to be −0.00678, 0.00727, −0.00362, and 5.19592 × 10^−4^, respectively. The fitting results are shown in Figure 7.

### 3.3. Model Validation

**Figure 8:**
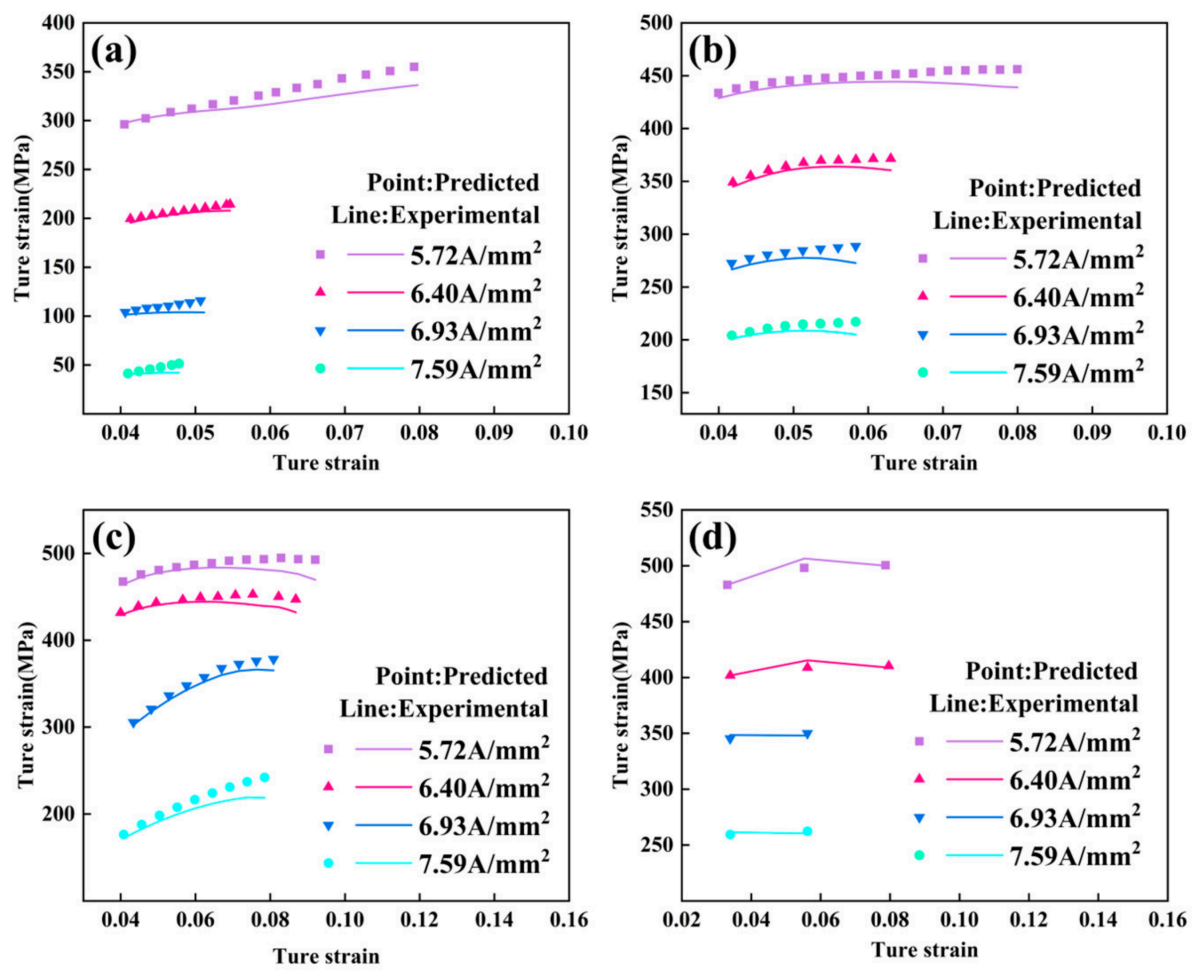
Comparison of experimental stress data with J-C model predictions at different strain rates and current densities: (**a**) 0.001 s^−1^; (**b**) 0.005 s^−1^; (**c**) 0.01 s^−1^; (**d**) 0.05 s^−1^.

A comparison of the experimental results with the predicted results is shown in Figure 8. The predictive capability of the constitutive equations was evaluated using the statistical parameters R and $R_{AARE}$ [26], which are calculated as follows:

(9) $$R=\frac{\sum_{i}^{N}(P_{exp}-{\bar{P}}_{exp})(P_{P}-{\bar{P}}_{P})}{\sqrt{\sum_{i}^{N}(P_{exp}-{\bar{P}}_{exp}{)}^{2}\sum_{i}^{N}(P_{P}-{\bar{P}}_{P}{)}^{2}}}\;\;\;\;$$

(10) $$R_{AARE}=\frac{1}{N}\sum_{i}^{N}\left|\frac{P_{p}-P_{exp}}{P_{exp}}\right|\;\;\;$$

**Figure 9:**
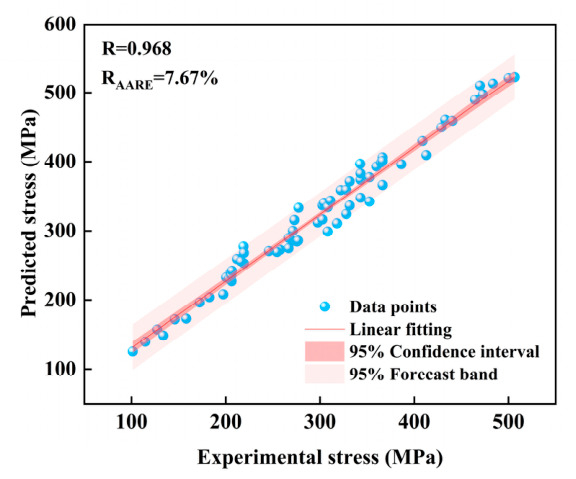
Correlation between measured and predicted actual stress values.

Here, $P_{exp}$ and ${\bar{P}}_{exp}$ denote the experimental stress and its average, respectively, while $P_{p}$ and ${\bar{P}}_{P}$ denote the predicted stress and its average, respectively; N represents the total number of observations. Figure 9 shows the correlation between the measured and predicted values of the true stress. Calculations yielded a correlation coefficient R of 0.968 and a mean absolute relative error $R_{AARE}$ of 7.67%, indicating that the constitutive model has good predictive capability.

It should be explicitly noted that the constitutive model established in this study is strictly calibrated within the following processing condition ranges: current density of 5.72–7.59 A/mm^2^, temperature of 550–780 °C, and strain rate of 0.001–0.05 s^−1^. Application of the model outside this range requires cautious validation of its predictive accuracy or recalibration of the relevant parameters.

## 4. Finite Element Simulation Analysis

### 4.1. Finite Element Modeling and Validation

**Figure 10:**
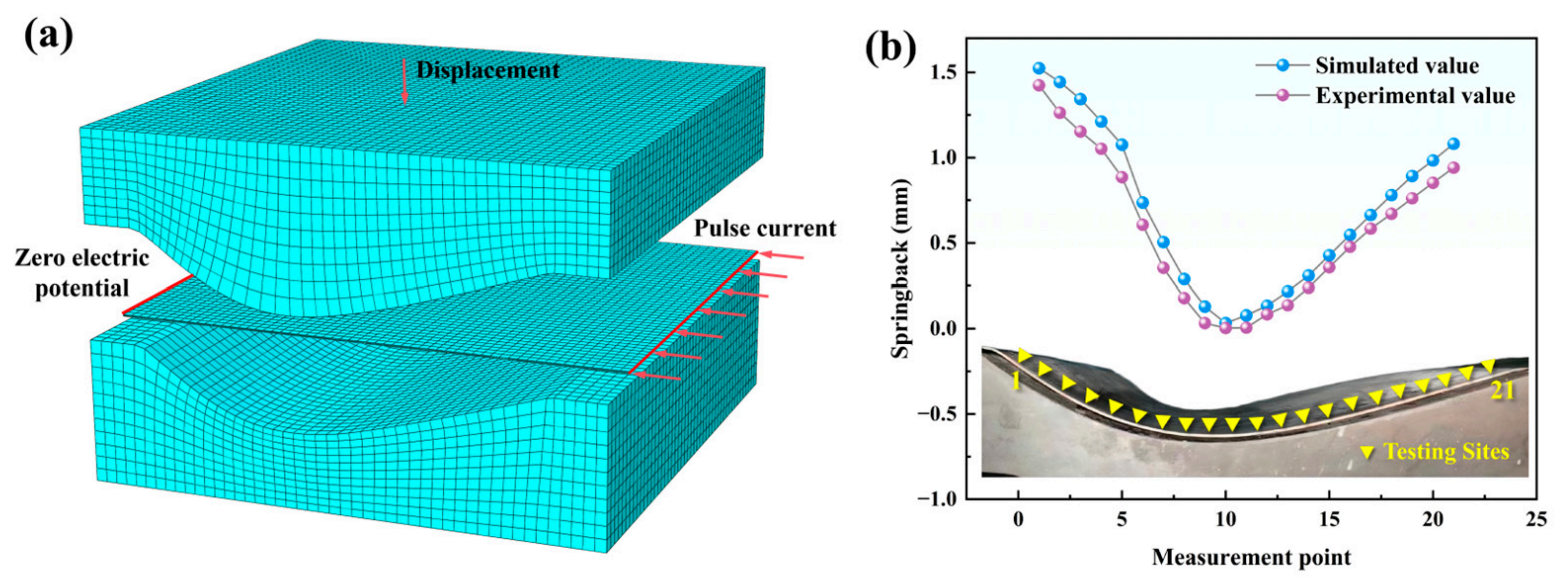
(**a**) Finite element model; (**b**) Comparison of form-fit accuracy between finite element simulation and forming experiments.

The electrically assisted forming process of Ti-6Al-4V alloy skin components was simulated using the ABAQUS (v2023) finite element software, with the finite element model depicted in Figure 10a. Material properties were defined based on the established constitutive model, and a coupled thermal-electrical-structural analysis step was employed. Since the dies were electrically insulated in the actual experiments, the electrical energy dissipation caused by current flowing through the dies was neglected. Electrical loading was applied by prescribing a surface current density on one end of the sheet and a zero electric potential on the other end. During the forming process, full fixation constraints were applied to the die, while a velocity load was prescribed to the punch to realize the stamping operation. A restart analysis was adopted to remove the contact interactions between the sheet and the dies as well as the external loads, thereby simulating the unloading and springback process.

To validate the reliability of the finite element model, electrically assisted forming experiments on titanium alloy skin components were conducted under the conditions of a current density of 7.59 A/mm^2^, a friction coefficient of 0.1, and a forming speed of 10 mm/min. The springback of the formed parts was measured using feeler gauges. The springback obtained from simulations and experiments is compared in Figure 10b. The relative error between the simulation and experimental results was 14.3%. This discrepancy can be attributed primarily to the following factors. In the actual experiments, the localized high-temperature effect induced by the electric current led to enhanced material ductility in the heated zones, thereby improving the die-fitting effect. In the simulation, however, a constant friction coefficient was adopted, whereas in practice the friction coefficient varies with temperature and contact pressure; localized high temperatures may cause a local increase in the friction coefficient, which in turn affects the springback. In addition, manual errors are inherent in the experimental measurement of springback using feeler gauges. Nevertheless, the simulated and experimental springback distributions exhibited consistent trends, and the relative error fell within the acceptable tolerance [27], confirming the reliability of the finite element model.

### 4.2. Temperature Distribution

**Figure 11:**
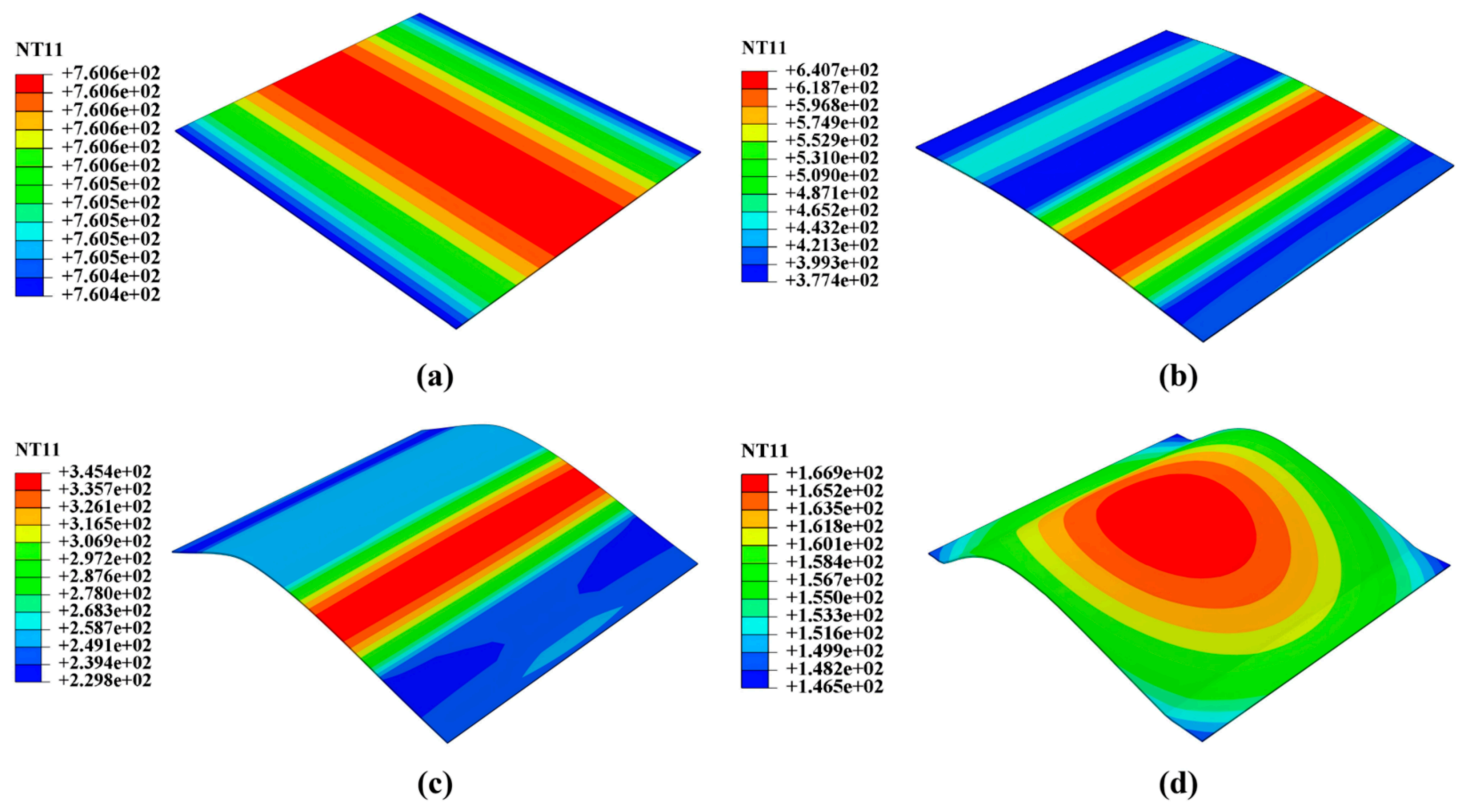
Temperature Distribution of the Blank at Different Stages of Forming: (**a**) Heating stage t = 120 s; (**b**) Bending stage t = 10 s; (**c**) Bending stage t = 20 s; (**d**) Bending stage t = 30 s.

The electrically assisted forming process was simulated under the conditions of a current density of 7.59 A/mm^2^, a friction coefficient of 0.1, and a forming speed of 10 mm/min. The temperature field distributions of the sheet at different forming stages are presented in Figure 11. After 120 s of electrical heating, the sheet temperature was uniformly distributed, with a maximum value of 760.6 °C. Subsequently, the punch began to move downward and came into contact with the sheet. The temperature in the contact regions decreased at a faster rate than that in the non-contact regions. At t = 10 s, the temperature in the contact regions dropped to 377.4 °C, while that in the non-contact regions remained at 640.7 °C. As the punch continued to advance, the sheet was progressively forced into contact with the die, leading to a further decrease in temperature. After the punch and die were completely closed, the temperature in the forming zone of the sheet decreased to 166.9 °C.

### 4.3. Stress Distribution

**Figure 12:**
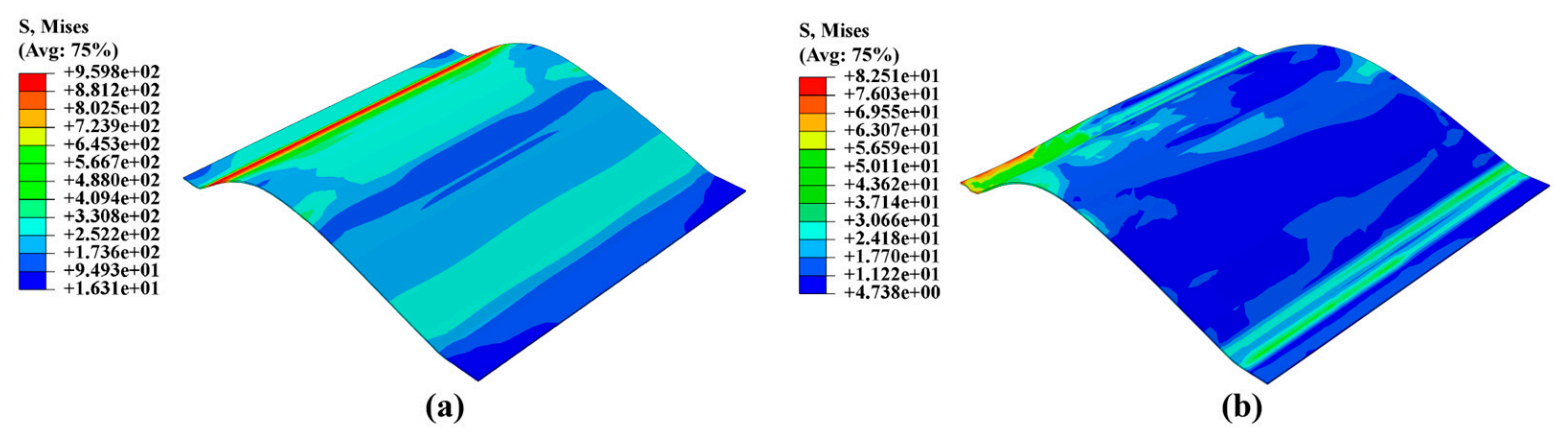
Comparison of Stress Distributions Before and After Rebound: (**a**) Before springback; (**b**) After springback.

Figure 12 presents a comparison of the stress distributions before and after springback. After electrically assisted bending forming, a pronounced stress concentration was observed in the formed component, with the left side of the bending zone exhibiting a high-stress region. Upon removal of the die constraints, the sheet underwent springback, during which the internal residual stresses were progressively released through elastic strain recovery. This process manifested as a marked reduction in the peak stress and the elimination of stress concentration. Concurrently, the stress distribution expanded from a banded pattern to a more laterally distributed pattern across the sheet, resulting in an overall more uniform stress state.

### 4.4. Effect of Current Density on Springback

**Figure 13:**
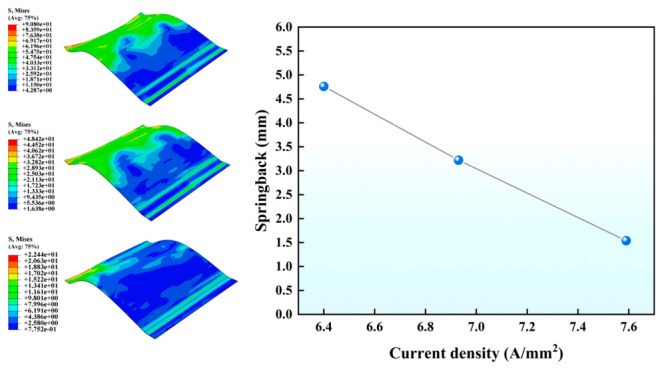
Variation in springback with current density.

The current density directly determines the electrical heating temperature of the sheet blank, i.e., the initial forming temperature. Furthermore, during the forming process, heat transfer between the sheet and the dies leads to a temperature decrease in the sheet, whereas the electric current compensates for this heat loss to a certain extent, thereby influencing the overall forming temperature. Figure 13 presents the maximum springback of the formed parts under different current densities, at a friction coefficient of 0.1 and a forming speed of 10 mm/min.

With increasing current density, the die-fitting accuracy of the formed parts improved progressively, which is primarily attributable to the elevated forming temperature resulting from the increased current density. When the current density was increased from 6.40 A/mm^2^ to 7.59 A/mm^2^, the electrical heating temperature of the sheet rose from 613.2 °C to 760.6 °C, and the springback decreased from 4.76 mm to 1.54 mm. Increasing the current density can effectively suppress springback. However, in practical forming operations, the potential degradation of material properties induced by elevated temperatures must be taken into account. Therefore, the selection of an appropriate current density should be based on a comprehensive consideration of the overall forming quality.

### 4.5. Effect of Forming Speed on Springback

**Figure 14:**
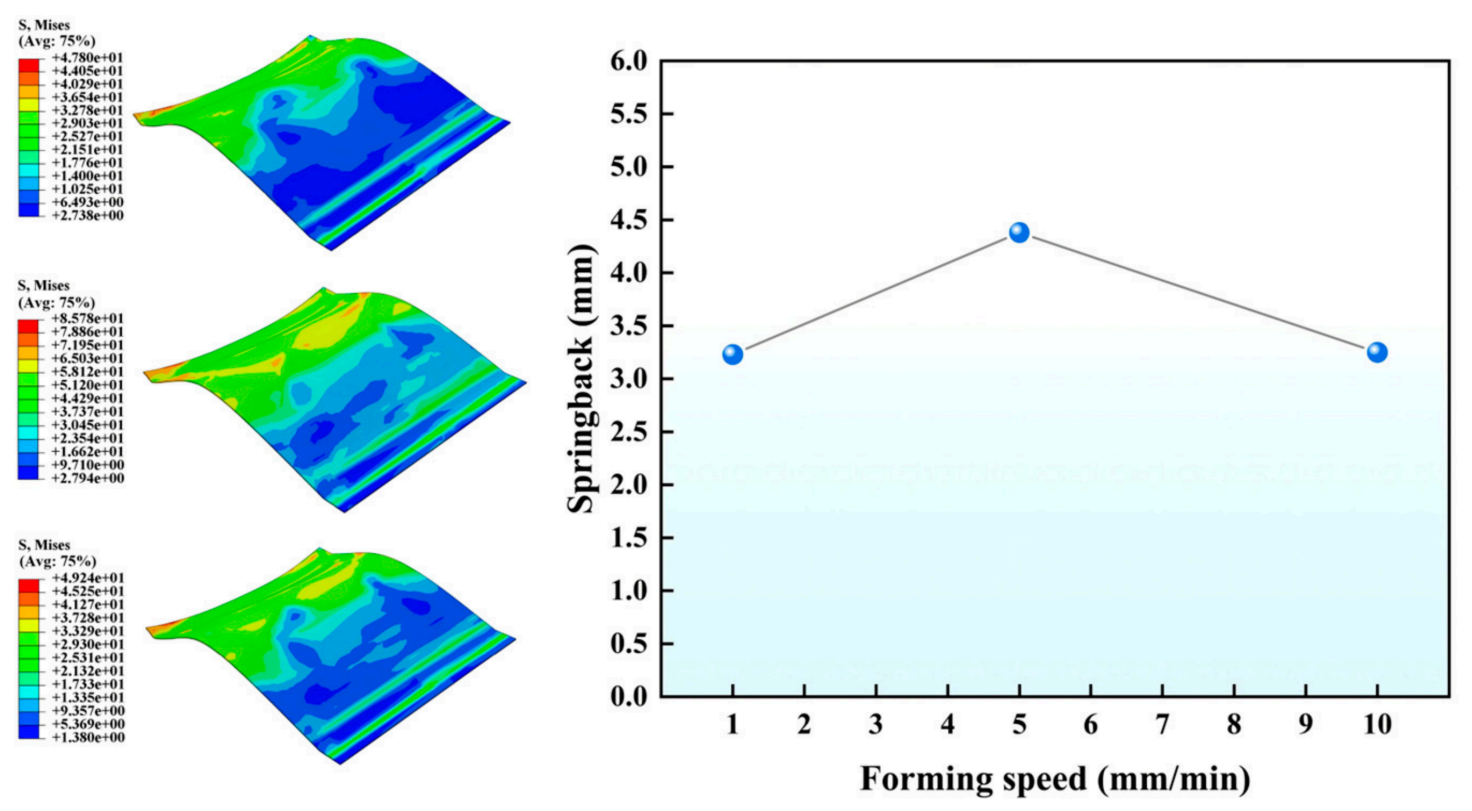
Variation in springback with forming speed.

Electrically assisted forming is a cold-die hot-forming process, in which the sheet blank is heated to a high temperature while the dies remain at room temperature. During the forming operation, heat transfer occurs between the sheet and the dies; consequently, the forming speed is a critical factor influencing springback. Figure 14 presents the maximum springback of the formed parts at different forming speeds, with a friction coefficient of 0.1 and a current density of 6.93 A/mm^2^. With increasing forming speed, the springback of the formed parts exhibited a trend of first increasing and then decreasing. Specifically, as the forming speed increased from 1 to 5 mm/min, the springback increased gradually, with a notable surge observed at 5 mm/min. However, when the forming speed was further raised to 10 mm/min, the springback decreased. This reduction is primarily attributable to the weakened heat conduction at higher forming speeds, where the electrical current provides thermal compensation, resulting in a higher forming temperature of the sheet. Under such conditions, the material exhibits improved ductility compared with that at lower forming speeds, leading to increased plastic deformation under the same level of deformation.

### 4.6. Effect of Friction Coefficient on Springback

**Figure 15:**
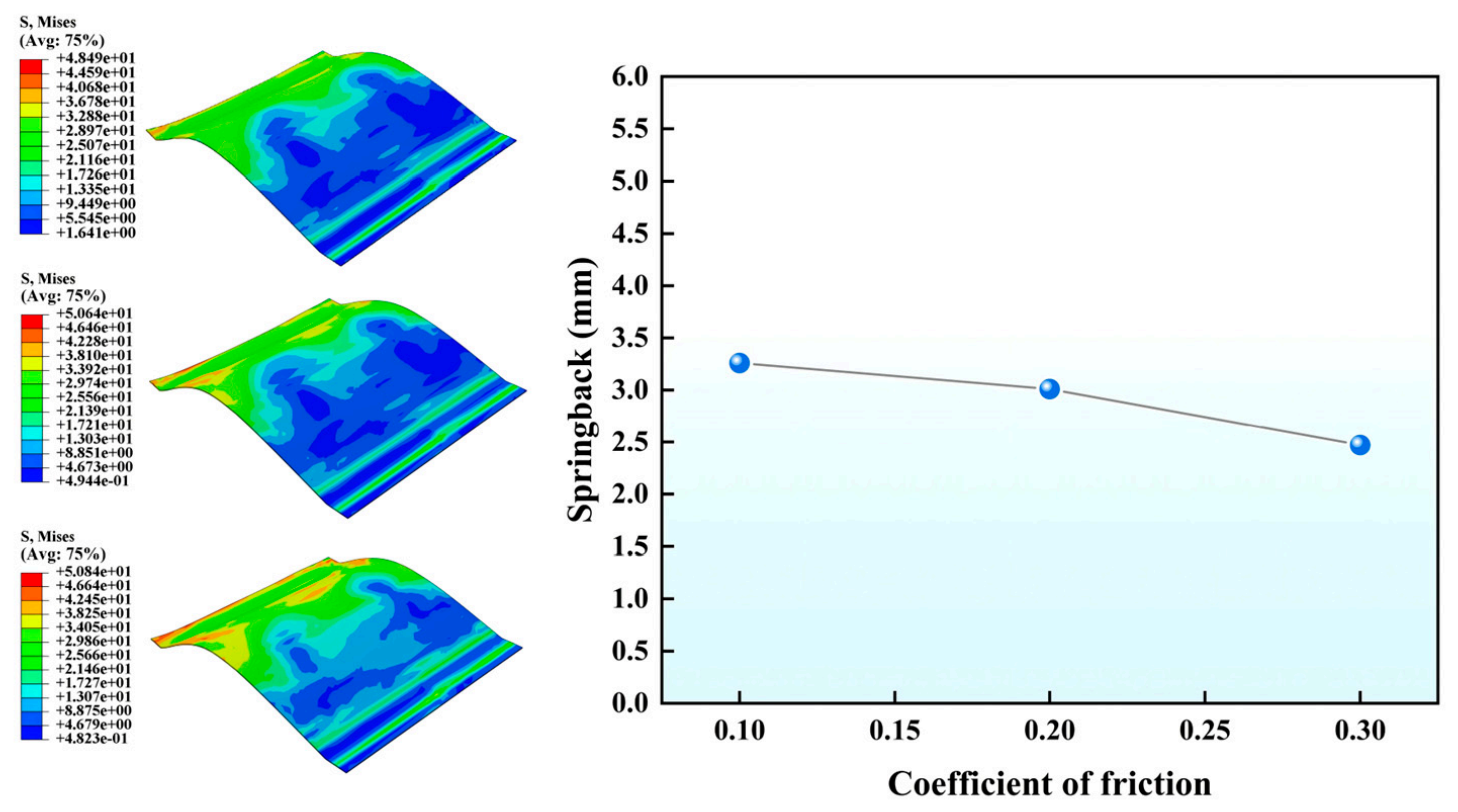
Variation in springback with friction coefficient.

The friction condition between the sheet blank and the dies governs the material flow behavior, which in turn affects the forming quality of the final component. Therefore, in hot forming processes, proper control of the friction coefficient serves as an effective means of regulating the forming quality. Figure 15 presents the maximum springback of the formed parts under different friction coefficients, at a forming speed of 10 mm/min and an equivalent current density of 6.93 A/mm^2^. With increasing friction coefficient, the springback of the formed parts exhibited a slightly decreasing trend, though the reduction was not significant. At higher friction coefficients, the compressive stress at the contact interface between the sheet and the dies increases, which enhances the tensile stress component in the bending deformation zone. The induced tensile stress causes more material to undergo plastic deformation beyond the elastic strain limit. Consequently, the neutral layer shifts from the sheet thickness center toward the compression side, accompanied by an expansion of the plastic deformation region and a corresponding reduction in the proportion of elastic deformation, thereby suppressing springback. However, an excessively high friction coefficient may lead to issues such as sheet cracking, poor surface quality of the part, and accelerated die wear. Therefore, in practical forming operations, it is desirable to maintain the friction coefficient as low as possible to ensure satisfactory component quality.

## 5. Experimental Setup and Procedure

### 5.1. Electrically Assisted Experimental Apparatus

**Figure 16:**
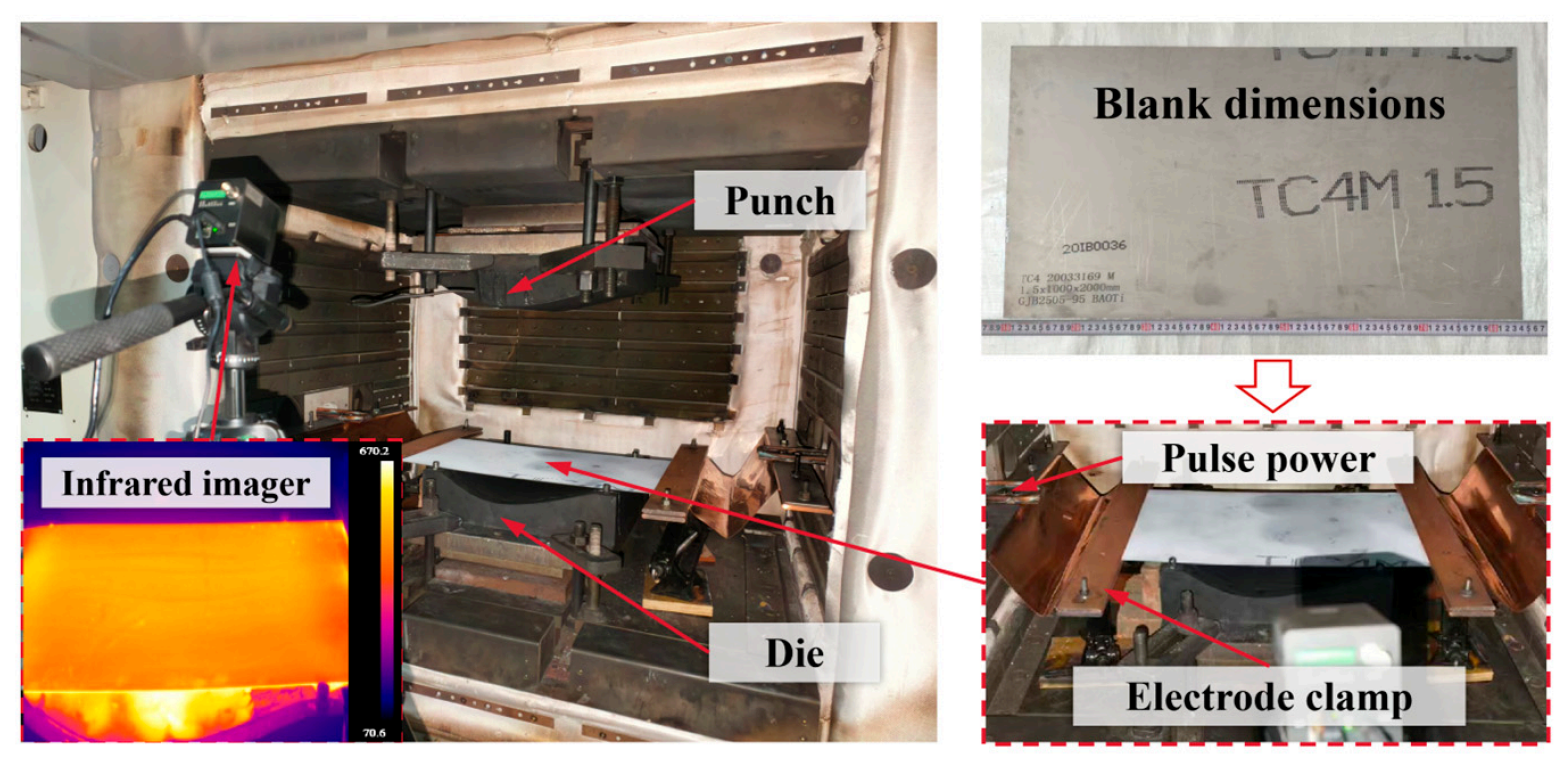
Electrically assisted forming setup.

An electrically assisted forming setup was established based on the HT-ER-210T CNC gas blow forming equipment, as shown in Figure 16. The experimental apparatus consists of a metallic punch, a metallic die, copper electrode clamping plates, and bakelite sheets. The pulsed power supply and the infrared thermal imager were consistent with those used in the electrically assisted tensile tests, serving for sheet heating and temperature monitoring, respectively. Bakelite sheets were employed to achieve electrical insulation between the dies and the worktable, while insulating varnish was applied between the sheet blank and the dies to prevent current dissipation caused by direct contact between the sheet and the dies.

During the electrically assisted forming experiments on thin-walled titanium alloy components, the sheet surface was uniformly coated with boron nitride lubricant, which exhibits a friction coefficient of approximately 0.08–0.12 in a dry environment at temperatures ranging from 400 °C to 800 °C. The sheet was then placed onto the die and connected to the pulsed power supply via copper electrode clamping plates. To minimize the influence of contact resistance and its fluctuations on localized heating conditions [28], the surfaces of the copper electrode clamping plates were precision ground. The electrode clamping force was precisely controlled using a torque wrench, and the contact surfaces were cleaned with acetone in an ultrasonic bath prior to each experiment to ensure consistency of the contact conditions. The sheet was heated to a steady-state temperature by the electric current. While the current remained applied, the punch moved downward until die closure was achieved, followed by a holding stage with current application and subsequent pressure release with the current turned off. Finally, the die was opened, the formed part was removed, and the excess material was trimmed to obtain the target component.

### 5.2. Experimental Study on Electrically Assisted Forming

**Figure 17:**
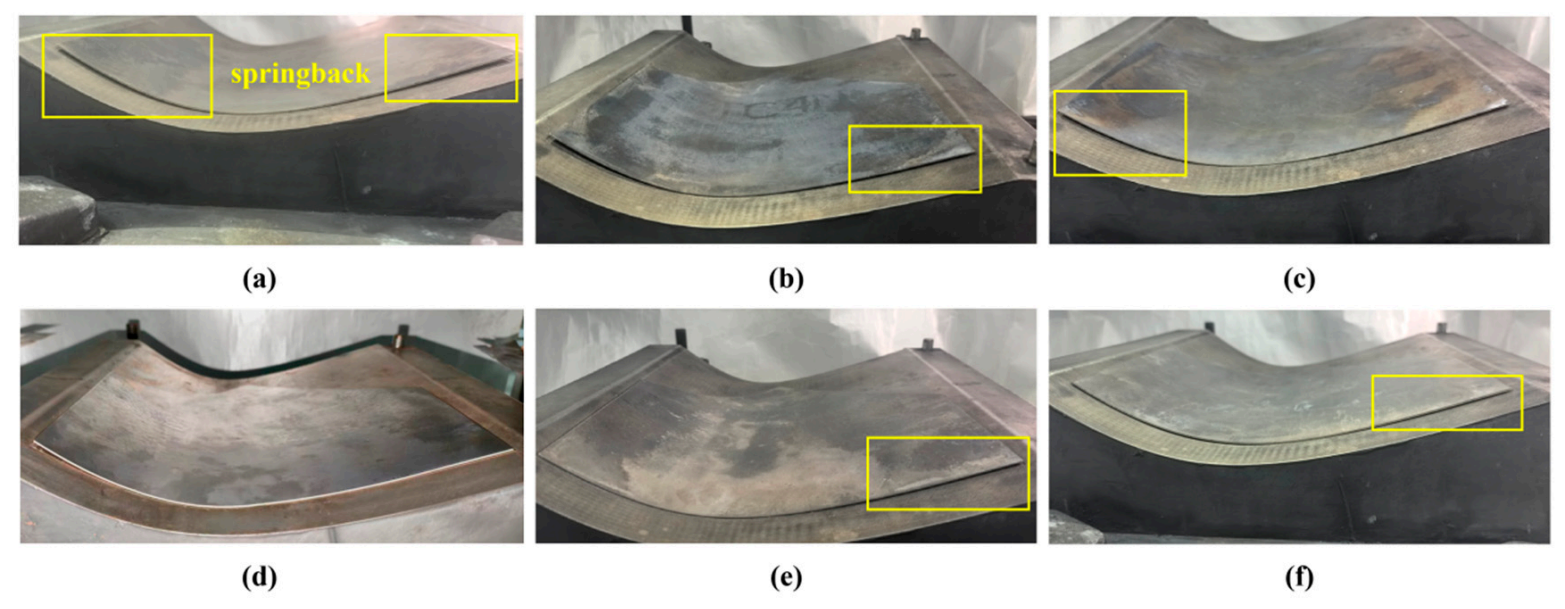
Thin-walled titanium alloy components under different forming conditions: (**a**) 6.40 A/mm^2^, 5 mm/min, uninsulated; (**b**) 6.40 A/mm^2^, 5 mm/min; (**c**) 6.93 A/mm^2^, 5 mm/min; (**d**) 7.59 A/mm^2^, 1 mm/min; (**e**) 7.59 A/mm^2^, 5 mm/min; (**f**) 7.59 A/mm^2^, 10 mm/min.

To investigate the effects of different forming conditions on the formability of thin-walled titanium alloy components, electrically assisted forming experiments were conducted at a friction coefficient of 0.1, with current densities of 6.40 A/mm^2^, 6.93 A/mm^2^, and 7.59 A/mm^2^ and forming speeds of 1 mm/min, 5 mm/min, and 10 mm/min. At least three repeated experiments were performed for each process condition. Figure 17a,b show the formed components under different insulation conditions at the same current density and forming speed. Without the application of insulating varnish, current transferred from the sheet to the die, resulting in a reduced electrical heating temperature of the sheet and consequently severe springback after forming. With the application of insulating varnish, the springback of the formed components was significantly suppressed. Figure 17b,c,e present the formed components at different current densities with a forming speed of 5 mm/min. As the current density increased, the electrical heating temperature of the sheet rose from 606.5 °C to 754.6 °C, accompanied by a gradual decrease in springback. Increasing the current density elevates the electrical heating temperature of the sheet, thereby ensuring a higher forming temperature, enhancing material ductility, and consequently suppressing springback. Figure 17d–f show the components formed at different forming speeds with a current density of 7.59 A/mm^2^. With increasing forming speed, the springback first increased and then decreased. At a forming speed of 1 mm/min, the component exhibited good surface quality and a maximum springback of 1.04 mm. Therefore, reasonable matching of current density and forming speed is essential in the electrically assisted forming of thin-walled titanium alloy components to improve formability and mitigate springback defects.

### 5.3. Mechanical Properties and Microstructure

**Figure 18:**
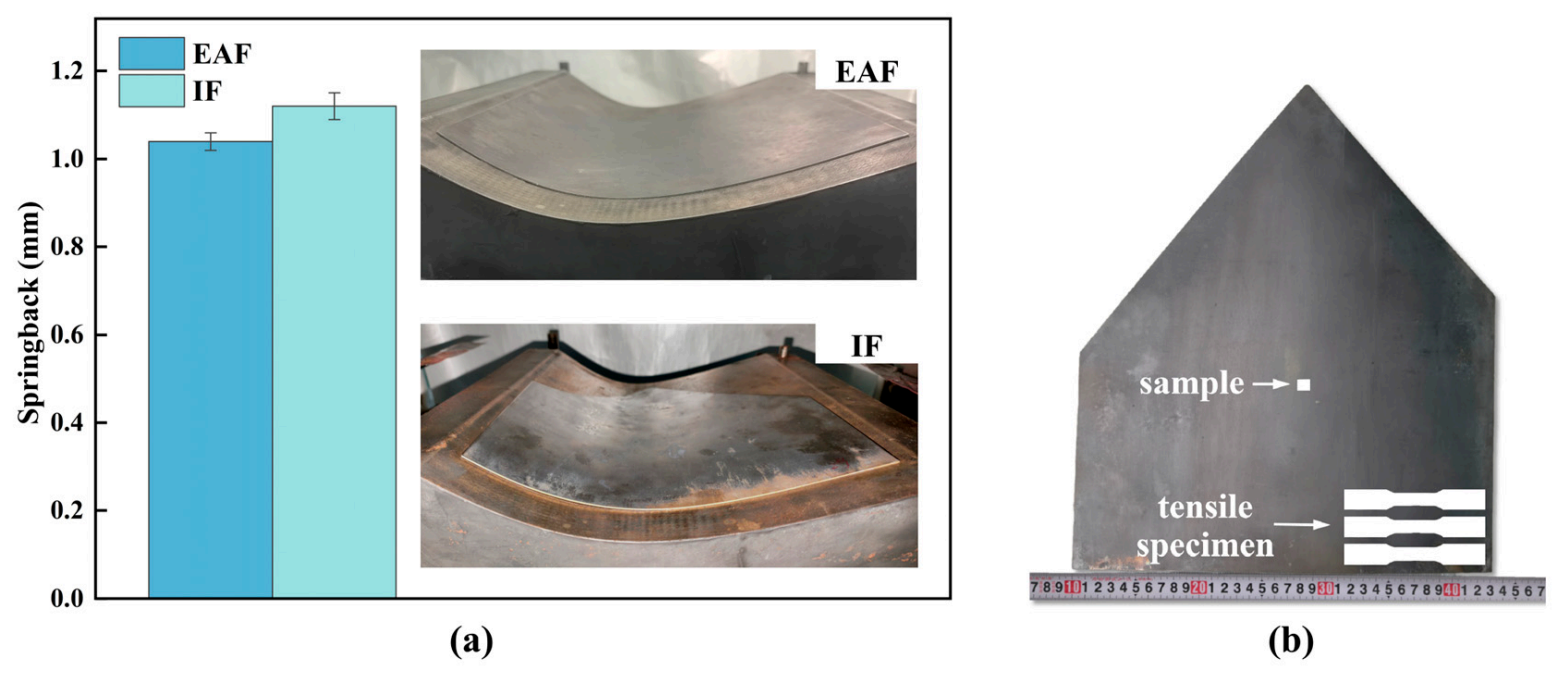
(**a**) Comparison of parts formed by EAF and IF; (**b**) Microstructure and tensile specimen extraction locations.

To investigate the differences between EAF and IF, comparative experiments were conducted, as shown in Figure 18a. For the EAF process, a current density of 7.59 A/mm^2^, a forming speed of 1 mm/s, and a friction coefficient of 0.1 were selected. For the IF process, a forming temperature of 750 °C, a forming speed of 1 mm/s, and a friction coefficient of 0.1 were adopted. The experimental results revealed that the maximum springback of the EAF-formed component was 1.04 mm, whereas that of the IF-formed component was 1.12 mm. This difference in springback is closely related to the distinct temperature field distributions characteristic of the two processes. In the IF process, the uniform temperature field leads to a consistent degree of thermal softening throughout the material; consequently, the elastic recovery after unloading is primarily governed by the overall deformation magnitude and the yield strength. In the EAF process, however, Joule heat is generated internally within the sheet. The inhomogeneous heat dissipation conditions give rise to a non-uniform temperature field during forming, with the central deformation zone reaching a higher temperature. This elevated temperature reduces the flow stress and yield strength, inducing localized softening that facilitates deformation concentration within the forming region. To further evaluate the forming quality of the two processes, the mechanical properties and microstructure of the formed components were investigated. Tensile specimens and electron backscatter diffraction (EBSD) specimens were both extracted from the same deformation-featured zones of the components formed by the two processes. Each tensile test was repeated three times to ensure reliability, as illustrated in Figure 18b.

**Figure 19:**
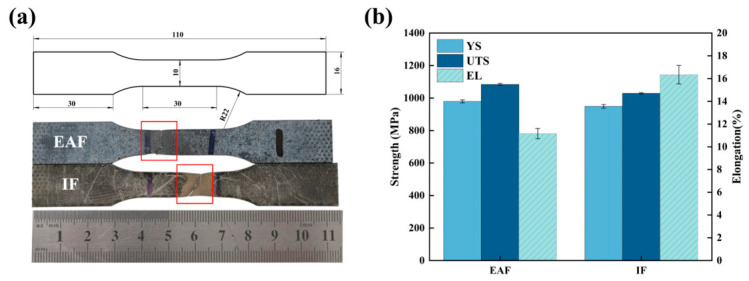
(**a**) Tensile fracture location; (**b**) Comparison of tensile properties between EAF and IF parts.

Figure 19 presents a comparison of the tensile properties between the EAF- and IF-formed components. The EAF specimens exhibited a yield strength (YS) of 979.8 MPa, an ultimate tensile strength (UTS) of 1084.5 MPa, and an elongation (EL) of 11.17%. In comparison, the IF specimens showed a yield strength of 949.1 MPa, an ultimate tensile strength of 1029.5 MPa, and an elongation of 16.34%. The fracture of the EAF specimens occurred near the gauge edge, with inconspicuous necking, indicative of a brittle fracture characteristic. In contrast, the IF specimens fractured near the middle of the gauge length, exhibiting pronounced necking and greater overall elongation in the gauge section, which is consistent with a ductile fracture mode. The EAF process involves rapid heating of the material, followed by a rapid cooling stage upon current interruption, during which the β-phase grains transform into secondary α′-phase [14]. The formation of secondary α′-phase enhances the strength of the material but significantly reduces its ductility, leading to premature necking and fracture during tensile testing. In the IF process, however, the material is cooled within the furnace, allowing sufficient time for the β → (α + β)-phase transformation. This results in a more homogeneous microstructure and improved plastic deformation capability.

**Figure 20:**
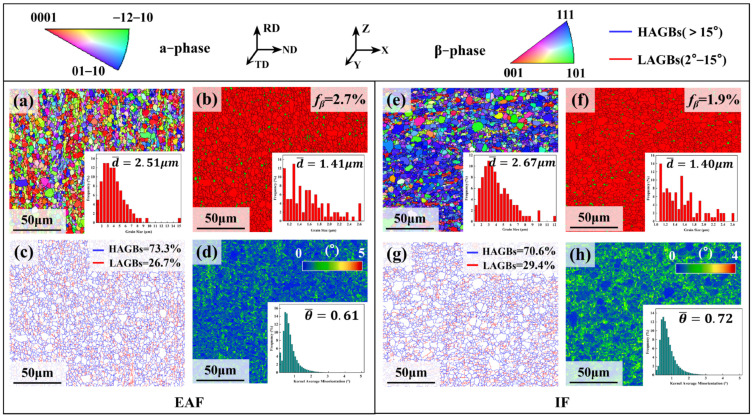
EBSD characterization results for EAF samples: (**a**) Inverse pole figure; (**b**) β-phase distribution map; (**c**) Grain boundary distribution map; (**d**) KAM map. EBSD characterization results for IF samples: (**e**) Inverse pole figure; (**f**) β-phase distribution map; (**g**) Grain boundary distribution map; (**h**) KAM map.

Figure 20 presents the EBSD characterization results of the EAF and IF samples, respectively. The EAF sample consists of equiaxed α grains and β-phases with a volume fraction of 2.7%, distributed along the grain boundaries. The average grain sizes of the α and β-phases are 2.51 μm and 1.41 μm, respectively. The IF sample consists of equiaxed α grains with 1.9% β-phase, and the average equivalent circle diameters of the α-phases and β-phases are 2.67 μm and 1.40 μm, respectively. As shown in Figure 20c,g, the grain boundaries of these equiaxed grains are composed of high-angle grain boundaries (HAGBs) and low-angle grain boundaries (LAGBs). The proportion of HAGBs in the EAF sample is 73.3%, slightly higher than that in the IF sample (70.6%). Regarding the kernel average misorientation (KAM) maps shown in Figure 20d,h, the EAF sample exhibits an average KAM value of 0.61°, with the KAM map appearing predominantly blue. In contrast, the IF sample exhibits an average KAM value of 0.72°, with more green regions. The geometrically necessary dislocation (GND) density can be quantitatively calculated from the KAM values using the following equation [29]:

(11) $$\rho^{GND}=\frac{2\bar{\theta }}{\mu b}$$

where $\rho^{GND}$ is the GND density, $\bar{\theta }$ is the average KAM value, μ is the step size used in the EBSD measurement (0.3 μm), and b is the magnitude of the Burgers vector of the α-phase in Ti–6Al–4V, taken as 0.295 nm [30]. The calculated average GND densities of the EAF and IF samples are 7.22 × 10^14^ m^−2^ and 8.52 × 10^14^ m^−2^, respectively. Compared with the IF sample, the GND density of the EAF sample is reduced by 15.3% [31].

The higher β-phase fraction in the EAF sample compared with that in the IF sample indicates that the pulsed current promotes the α → β-phase transformation in Ti-6Al-4V alloy through the coupled effects of Joule heating and athermal electroplastic effects [11,32], thereby reducing the flow stress of the material. This facilitates plastic flow during forming and enables better conformity of the sheet to the die cavity. Previous studies have suggested that an increased fraction of HAGBs is indicative of the occurrence of recrystallization to a certain extent [33]. The pulsed current promoted recrystallization of Ti-6Al-4V alloy, which refines the grain size and renders the grain distribution more uniform. This process eliminates a substantial amount of deformed microstructures and dislocation tangles, effectively alleviating the tendency for strain localization. Concurrently, the reduced GND density in the EAF sample suggests that the pulsed current decreases the overall dislocation density, thereby reducing the elastic strain energy stored within the deformed material and consequently diminishing the driving force for elastic recovery upon unloading.

## 6. Discussion

This study aims to validate the feasibility of the EAF process in forming thin-walled Ti-6Al-4V alloy skin components, thereby filling the research gap concerning the application of this process in the field of aerospace thin-walled components. Compared with IF, the EAF process adopted in this work realized the integration of heating and forming for titanium alloy skin components, significantly reduced energy consumption, and improved production efficiency by more than threefold. In addition, the EAF process avoids die heating, which extends die service life and eliminates the need for expensive heat-resistant die materials, thereby reducing production costs. The forming equipment is also simplified, as heating rods and thermal insulation facilities are no longer required [34]. In this study, the springback of the skin component was reduced by 7.14%, the ultimate tensile strength was increased by 5.34%, while the elongation was reduced by 31.64%. The microstructure of the material was also refined, demonstrating promising application prospects. Although the elongation decreased, the elongation of the EAF specimens still met the engineering application requirement of greater than 10% [35]. It should be noted that the mechanical properties reported in this study were obtained without any post-forming heat treatment. In actual industrial production, the ductility of the material can be further improved by introducing a short-duration solution treatment and aging. Moreover, the electroplastic effect is influenced by the current density, frequency, and duty cycle of the pulsed current. The pulse frequency affects the mechanical properties and microstructure of the material, and under certain frequencies, the material may exhibit superior mechanical performance [36]. By optimizing the duty cycle and adjusting the pulse interval time, the athermal effect at specific duty cycles can more effectively reduce the resistance to dislocation motion, thereby promoting more uniform deformation [37]. Therefore, future work will focus on investigating the effects of different electrical parameters through well-designed single-variable experiments to optimize the pulsed current parameters for improved formability. Furthermore, multi-scale microstructural characterization using EBSD and TEM will be conducted to further elucidate the underlying micro-mechanisms of electrically assisted forming of thin-walled Ti-6Al-4V alloy skin components.

## 7. Conclusions

In this paper, an EAF process is proposed for the fabrication of thin-walled Ti-6Al-4V alloy skin components, aiming to provide a new strategy for the high-performance manufacturing of thin-walled titanium alloy components. To verify the feasibility of the EAF process, finite element simulations and experimental investigations were conducted. The following conclusions can be drawn:

(1) During the electrically assisted tensile deformation of Ti-6Al-4V alloy, the peak stress decreased progressively with increasing current density and decreasing strain rate. A constitutive model was established based on the Johnson–Cook framework, with a correlation coefficient (R) of 0.968 and an average absolute relative error (R_AARE_) of 7.67%, indicating that the model can effectively describe the stress–strain behavior of Ti-6Al-4V alloy.
(2) In the electrically assisted bending forming of Ti-6Al-4V alloy, the springback decreased gradually with increasing current density and friction coefficient, while it exhibited a trend of first increasing and then decreasing with increasing forming speed. At a current density of 7.59 A/mm^2^, a forming speed of 1 mm/min, and a friction coefficient of 0.1, the formed parts achieved relatively satisfactory performance, with a maximum springback of 1.04 mm.
(3) Under the same process conditions, the EAF-formed component exhibited a 7.14% reduction in springback and a 5.34% increase in ultimate tensile strength compared with the IF-formed component. EBSD characterization revealed that the pulsed current promoted recrystallization of the Ti-6Al-4V alloy, refined the grain structure, facilitated the α → β-phase transformation, and reduced the overall dislocation density. These findings validate the feasibility of the EAF process for achieving high-efficiency and high-precision forming of thin-walled titanium alloy components, and demonstrate its potential as a viable alternative to the conventional IF process.

## Figures and Tables

**Table 1:** Chemical composition of Ti-6Al-4V alloy (%, by mass).

Al	V	Fe	O	Ti
5.88	3.95	0.3	0.1	Bal.

## Data Availability

The original contributions presented in this study are included in the article. Further inquiries can be directed to the corresponding author.
